# High prevalence of multidrug resistant *Enterobacteriaceae* isolated from outpatient urine samples but not the hospital environment in Bo, Sierra Leone

**DOI:** 10.1186/s12879-016-1495-1

**Published:** 2016-04-18

**Authors:** Tomasz A. Leski, Chris R. Taitt, Umaru Bangura, Michael G. Stockelman, Rashid Ansumana, William H. Cooper, David A. Stenger, Gary J. Vora

**Affiliations:** Naval Research Laboratory, Code 6910, 4555 Overlook Avenue SW, Washington, DC 20375 USA; Mercy Hospital Research Laboratory, Bo, Sierra Leone; Liverpool School of Tropical Medicine, Liverpool, UK; Njala University, Bo, Sierra Leone; Bowie State University, Bowie, Maryland USA

**Keywords:** *Citrobacter*, MDR, ESBL, CHROMagar, Sierra Leone

## Abstract

**Background:**

The rising level of antimicrobial resistance among bacterial pathogens is one of the most significant public health problems globally. While the antibiotic resistance of clinically important bacteria is closely tracked in many developed countries, the types and levels of resistance and multidrug resistance (MDR) among pathogens currently circulating in most countries of sub-Saharan Africa are virtually unknown.

**Methods:**

From December 2013 to April 2014, we collected 93 urine specimens from all outpatients showing symptoms of urinary tract infection (UTI) and 189 fomite swabs from a small hospital in Bo, Sierra Leone. Culture on chromogenic agar combined with biochemical and DNA sequence-based assays was used to detect and identify the bacterial isolates. Their antimicrobial susceptibilities were determined using a panel of 11 antibiotics or antibiotic combinations.

**Results:**

The 70 *Enterobacteriaceae* urine isolates were identified as *Citrobacter freundii* (*n* = 22), *Klebsiella pneumoniae* (*n* = 15), *Enterobacter cloacae* (*n* = 15), *Escherichia coli* (*n* = 13), *Enterobacter* sp./*Leclercia* sp. (*n* = 4) and *Escherichia hermannii* (*n* = 1). Antimicrobial susceptibility testing demonstrated that 85.7 % of these isolates were MDR while 64.3 % produced an extended-spectrum ß-lactamase (ESBL). The most notable observations included widespread resistance to sulphonamides (91.4 %), chloramphenicol (72.9 %), gentamycin (72.9 %), ampicillin with sulbactam (51.4 %) and ciprofloxacin (47.1 %) with *C. freundii* exhibiting the highest and *E. coli* the lowest prevalence of multidrug resistance. The environmental cultures resulted in only five *Enterobacteriaceae* isolates out of 189 collected with lower overall antibiotic resistance.

**Conclusions:**

The surprisingly high proportion of *C. freundii* found in urine of patients with suspected UTI supports earlier findings of the growing role of this pathogen in UTIs in low-resource countries. The isolates of all analyzed species showed worryingly high levels of resistance to both first- and second-line antibiotics as well as a high frequency of MDR and ESBL phenotypes, which likely resulted from the lack of consistent antibiotic stewardship policies in Sierra Leone. Analysis of hospital environmental isolates however suggested that fomites in this naturally ventilated hospital were not a major reservoir for *Enterobacteriaceae* or antibiotic resistance determinants.

**Electronic supplementary material:**

The online version of this article (doi:10.1186/s12879-016-1495-1) contains supplementary material, which is available to authorized users.

## Background

The increasing rate of antibiotic resistance among bacterial pathogens causing both hospital- and community-acquired infections is a serious threat to public health worldwide [1, 2]. Trends among pathogens belonging to the *Enterobacteriaceae* are especially troubling considering their ubiquity in the environment and animal hosts and the relative ease with which they acquire and transfer genetic determinants that confer resistance to most classes of antibiotics. Recently, this phenomenon has resulted in the emergence and international spread of bacteria causing a number of deadly hospital outbreaks [3, 4]. These include increasingly abundant and problematic bacterial strains harboring extended-spectrum ß-lactamases (ESBL) that confer resistance to the majority of ß-lactam antibiotics and carbapenemases that confer resistance to carbapenem compounds, the most powerful and last line of ß-lactam antibiotics [5–9]. Typically, ESBL-producing strains, carbapenem resistant *Enterobacteriaceae* (CRE), and other multidrug resistant (MDR) *Enterobacteriaceae* possess several additional resistance mechanisms to other classes of popular antibiotics such as phenicols, sulfonamides, fluoroquinolones, tetracyclines, and aminoglycosides [10, 11]. This makes them extremely difficult – and in some cases, virtually impossible – to treat.

While MDR bacterial pathogens have been vigorously investigated in countries with mandated antibiotic stewardship policies and adequate resources, scant data are available from developing countries [12]. As a salient example, a recent survey of the antimicrobial susceptibility of *Enterobacteriaceae* that caused urinary tract infections (UTI) in Africa revealed that <200 studies were published between the years 2000 and 2012. Furthermore, although these analyses originated from 14 African countries, 90 % of the analyzed isolates came from South Africa and only a handful of the studies were conducted in other sub-Saharan African countries [13]. This kind of data is critical for developing viable antibiotic use policies and therapeutic strategies, especially since these same antimicrobial compounds are available without prescription in many developing countries (often resulting in widespread misuse [14–16]) and the situation can be further confounded by the presence of counterfeit or substandard quality drugs [17, 18]. To date, no official data on antimicrobial resistance of *Enterobacteriaceae* circulating in Sierra Leone are available [12]. To begin to gain some insight into the antibiotic sensitivity of *Enterobacteriaceae* in this area, we conducted a prospective study at Mercy Hospital (Bo, Sierra Leone) using bacterial isolates obtained from urine of patients with symptoms consistent with UTI and the samples collected from the surrounding hospital environment.

## Methods

### Bacterial isolates from clinical and hospital environmental samples

Mercy Hospital is a small private hospital in Bo, Sierra Leone, that refers about 2000 patients per year to its own clinical laboratory for testing [19]. Clinical samples for this study were obtained between December 12th 2013 and January 27th 2014 from all outpatients showing symptoms of UTI and referred for urine culture by the attending clinician. The samples originated from patients ranging in age from 1 to 59 with 63.4 % of the patients being female.

A total of 93 urine samples were screened on CHROMagar Orientation medium (CHROMagar, Paris, France). Seventy isolates were obtained from samples originating from 59 patients with significant bacteriuria (some samples yielded more than one isolate due to mixed infections). They included 57 isolates producing colonies with a dark blue color after overnight incubation at 37 °C (a phenotype characteristic of *Klebsiella*, *Enterobacter*, *Citrobacter* and *Serratia* when grown on this medium). Although this study was designed with the overall goal to analyze the antimicrobial resistance patterns of the non-*E. coli Enterobacteriaceae*, given the accepted role of *Escherichia coli* as the most prevalent bacterial pathogen causing UTI, 13 isolates producing dark pink to reddish colonies (the *E. coli* phenotype on this medium) were also added to the study.

Environmental isolates were obtained from 189 swab samples collected in and around the hospital between January 12th, 2014 and April 29th, 2014. The swabbing was performed using sterile cotton swabs moistened with sterile saline. Surfaces of approximately 10 cm^2^ of the tested area were swabbed and the swab was then swept across a CHROMagar Orientation medium plate which was subsequently incubated overnight at 37 °C. The areas swabbed included outpatient waiting areas, general and maternity wards, and waiting areas and benches in the diagnostic lab located in a separate building (Additional file 1: Table S1).

### Isolate identification

All collected *Enterobacteriaceae* isolates were identified to the species level using the API 20E identification system (bioMérieux, Marcy-l′Etoile, France) and 16S rRNA and *rpoB* gene sequencing. For the 16S rRNA-based identification, a fragment of 16S rDNA that spanned the V3 and V4 variable regions was PCR amplified and sequenced [20]. Genus- (or family-) level identification was determined using the naïve Bayesian classifier available through the Ribosomal Database Project (release 11) (http://rdp.cme.msu.edu/classifier/classifier.jsp), with 80 % confidence as the identification threshold [21]. Identification based on *rpoB* gene sequencing was performed using previously published primers [22, 23], with a BLAST search performed on the obtained amplicon DNA sequence. The highest-ranking BLAST result with the highest coverage was used as the basis for organism identification. A final consensus identification was based on a combination of the three identification methods (Table 1). In instances where the methods did not result in concordant identifications, a genus-level identification was assigned to the isolate based on the agreement of two of the methods. The species identification determined by *rpoB* gene sequencing was used when the phenotypic identification method and *rpoB* sequencing results were discordant. Clinical isolates for which API 20E-based identification was ambiguous (SL108, SL116, SL122, SL124, SL127) or for which the three identification methods gave discordant results (SL184, SL191, SL201 and SL204) were subjected to additional identification using the ID 32 rapid system (bioMérieux). Twenty-six of the non-*Enterobacteriaceae* environmental isolates were identified to the genus level only using 16S rRNA gene sequencing.

**Table 1:** Antibiotic sensitivity testing results

ID	Identification^a^	Disk diffusion^b^											E-test	ESBL^c^	
		ATM	CAZ	CIP	IMP	TGC	C	D	G	GM	K	SAM	IMP	Phenotype	CTX-M
Clinical isolates															
SL122	*Citrobacter freundii*	R	R	R	S	S	R	R	R	R	R	R	0.250	YES	YES
SL124		R	I	R	S	S	R	R	R	R	R	R	0.380	YES	YES
SL127		R	I	R	S	I	R	R	R	R	R	R	0.380	YES	YES
SL129		S	I	R	S	I	R	R	R	R	I	R	0.190	N/D	NO
SL150		R	R	R	S	I	I	R	R	R	R	R	0.190	YES	YES
SL151		R	I	R	S	S	R	R	R	R	I	R	0.380	YES	YES
SL153		R	R	R	I	I	R	R	R	R	I	R	0.250	YES	YES
SL154		R	R	R	I	S	R	R	R	R	I	R	0.190	N/D	NO
SL155		R	R	R	S	S	R	R	R	R	I	R	0.250	N/D	NO
SL156		R	R	R	I	I	R	I	R	R	R	R	0.250	YES	YES
SL157		R	R	R	S	I	R	I	R	R	R	R	0.250	YES	YES
SL170		R	I	R	S	S	R	R	R	R	I	R	0.750	YES	YES
SL172		R	R	R	S	S	S	R	R	R	R	I	0.190	YES	YES
SL173		R	R	R	I	S	R	R	R	R	I	R	0.380	YES	YES
SL177		R	R	R	S	S	R	R	R	R	I	R	0.250	YES	YES
SL187		R	I	R	S	S	S	I	R	R	I	I	0.250	YES	YES
SL193		R	I	R	S	S	R	I	R	R	S	R	0.250	YES	YES
SL194		R	R	R	S	S	R	R	R	R	I	R	0.380	N/D	NO
SL197		R	R	R	S	S	S	I	R	R	I	I	0.250	YES	YES
SL200		R	R	R	S	I	S	R	R	R	R	R	0.190	YES	YES
SL202		R	R	R	S	I	S	R	R	R	R	R	0.190	YES	YES
SL205		R	R	R	I	I	S	R	R	R	R	I	0.190	YES	YES
SL160	*Enterobacter cloacae*	S	S	S	S	S	S	S	R	S	S	S	0.380	NO	NO
SL161		R	R	I	I	I	R	S	R	R	R	R	0.250	YES	YES
SL167		I	I	I	S	S	R	I	R	R	I	R	0.380	YES	YES
SL168		R	R	I	I	S	R	I	R	R	I	R	2.000	YES	YES
SL174		R	I	I	S	S	R	I	R	R	R	I	0.380	YES	YES
SL175		I	I	S	S	S	R	S	R	R	R	R	0.250	YES	YES
SL179		R	R	R	S	S	R	I	R	R	R	R	0.250	YES	YES
SL181		S	S	S	S	S	R	S	R	R	I	I	0.380	YES	YES
SL182		R	R	I	I	S	R	S	R	R	I	R	0.380	YES	YES
SL189		S	S	I	S	S	R	S	R	R	I	I	0.250	YES	YES
SL192		S	S	I	S	S	R	R	R	R	S	S	0.380	NO	NO
SL195		I	I	S	S	S	R	S	R	R	I	I	0.380	YES	YES
SL198		R	R	S	S	S	R	S	R	R	I	R	0.250	YES	YES
SL199		R	I	I	S	S	R	S	R	R	I	I	0.380	YES	YES
SL203		I	I	I	S	S	R	S	R	R	I	I	0.380	YES	YES
SL115	*Escherichia coli*	S	S	S	S	S	S	S	S	S	S	S	0.190	NO	NO
SL121		S	S	R	S	S	R	R	R	S	S	I	0.190	NO	NO
SL125		S	S	S	S	S	S	S	S	S	S	S	0.190	NO	NO
SL133		S	S	S	S	S	S	R	R	S	S	R	0.190	NO	NO
SL137		S	S	S	S	S	S	R	R	S	S	R	0.190	NO	NO
SL152		S	S	I	S	S	R	R	R	R	S	S	0.190	NO	NO
SL158		S	S	R	I	S	R	R	R	S	S	R	0.190	NO	NO
SL159		S	S	S	I	S	S	S	S	S	S	S	0.190	NO	NO
SL169		S	S	S	S	S	R	R	R	S	S	S	0.250	NO	NO
SL171		S	S	S	S	S	R	I	R	S	S	R	0.250	NO	NO
SL176		S	S	R	S	S	S	S	R	S	S	I	0.250	NO	NO
SL178		R	S	S	S	S	R	I	R	I	S	S	0.250	NO	NO
SL188		S	S	S	S	S	R	I	R	S	S	S	0.250	NO	NO
SL166	*Escherichia hermannii*	R	R	I	S	S	R	S	R	R	I	R	0.190	YES	YES
SL108	*Klebsiella pneumoniae*	S	S	I	S	S	S	S	S	S	S	S	0.250	NO	NO
SL113		S	S	I	S	S	R	I	R	R	R	S	0.250	YES	YES
SL116		S	S	I	S	S	S	S	S	S	S	S	0.190	NO	NO
SL136		S	S	S	S	S	R	S	R	S	S	S	0.250	NO	NO
SL141		S	S	S	S	S	S	S	S	S	S	S	0.250	NO	NO
SL162		R	R	R	I	I	R	R	R	R	I	R	0.190	YES	YES
SL163		R	R	I	I	I	R	R	R	R	S	R	0.190	YES	YES
SL164		S	S	I	S	S	R	R	R	R	S	R	0.190	YES	YES
SL165		I	I	I	S	S	R	R	R	R	I	R	0.250	YES	YES
SL180		S	S	S	S	S	R	S	R	R	I	I	0.380	YES	YES
SL183		S	S	S	S	S	R	R	R	S	S	I	0.250	NO	NO
SL185		I	I	R	S	S	S	I	R	R	R	I	0.190	YES	YES
SL186		I	I	I	S	S	R	R	R	R	S	R	0.250	YES	YES
SL190		S	S	R	I	I	R	R	R	S	R	R	0.250	NO	NO
SL196		R	R	R	I	I	S	I	R	R	R	I	0.250	YES	YES
SL184	*Enterobacter* sp.*/Leclercia* sp.	I	I	R	S	S	R	S	R	R	I	I	0.250	YES	YES
SL191		I	R	R	I	I	R	S	R	R	I	I	0.250	YES	YES
SL201		R	R	R	R	I	R	S	R	R	R	I	0.250	YES	YES
SL204		R	R	I	R	I	R	R	R	R	R	I	0.190	YES	YES
Environmental isolates															
SL512	*Enterobacter cloacae*	S	S	S	I	S	S	R	R	S	S	S	0.250	NO	NO
SL532		I	I	S	S	S	R	S	R	R	R	R	N/A	YES	YES
SL513	*Klebsiella pneumoniae*	R	R	S	S	S	S	S	R	R	I	R	0.190	YES	YES
SL531		S	S	S	S	S	S	S	S	S	S	S	0.190	NO	NO
SL522	*Pantoea dispersa*	S	S	S	S	S	S	S	S	S	S	S	0.250	NO	NO

^a^Consensus identification based on combination phenotypic (API20E/ID32E) and 16S rRNA and *rpoB* gene sequencing

^b^Disk diffusion testing using the following antibiotics: *ATM* aztreonam, *CAZ* ceftazidime, *CIP* ciprofloxacin, *IMP* imipenem, *TGC* tigecycline, *C* chloramphenicol, *D* doxycycline, *G* sulfisoxazole, *GM* gentamicin, *K* kanamycin, *SAM* ampicillin/sulbactam. *R* resistant, *I* intermediate, and *S* sensitive phenotypes were determined based on CLSI criteria

^c^ESBL phenotypes: YES – ESBL detected, NO – no ESBL present, N/D – ESBL presence could not be determined based on the E-test or double disk diffusion assay

### Antibiotic susceptibility testing and ESBL detection

Every clinical and environmental isolate included in this study was assayed by disk diffusion for susceptibility to the following antibiotics or antibiotic combinations: ampicillin with sulbactam (SAM), aztreonam (ATM), ceftazidime (CAZ), chloramphenicol (C), ciprofloxacin (CIP), doxycycline (D), gentamicin (GM), imipenem (IMP), kanamycin (K), sulfisoxazole (G), and tigecycline (TGC). The susceptibility testing was conducted using BBL Mueller Hinton II Agar (MHA medium, Becton Dickinson, Franklin Lakes, NJ) according to Clinical and Laboratory Standards Institute (CLSI) recommendations [24]. CLSI interpretative criteria were applied for classification of the isolates into susceptible, intermediate and resistant categories [24]. In the case of tigecycline, for which there are no CLSI criteria, FDA recommended breakpoints were used [25]. Minimum inhibitory concentrations (MIC) for imipenem were determined using imipenem E-test strips (bioMérieux) according to manufacturer’s recommendations. The ESBL phenotype was determined using cefotaxime/cefotaxime + clavulanic acid (CT/CTL) and ceftazidime/ceftazidime + clavulanic acid (TZ/TZL) E-test strips (bioMérieux) according to manufacturer’s recommendations or by the disk diffusion method using disks containing cefotaxime/cefotaxime + clavulanic acid and ceftazidime/ceftazidime + clavulanic acid according to CLSI recommendations [24]. *E. coli* reference strain ATCC 25922, susceptible to all 11 antibiotics tested in this study, was used for quality control purposes. The MDR phenotype was defined as the demonstrated resistance to at least three antibiotics belonging to different classes of antimicrobial compounds.

### PCR-based detection of *bla*_CTX-M_

All of the isolates were tested for the presence of genes encoding CTX-M type ESBLs using universal CTX-M primers as previously described [26].

## Results

### Identification of isolates from urine cultures

Out of the 93 urine samples tested, 57 generated dark (metallic) blue colonies after an overnight incubation on CHROMagar Orientation medium, which was indicative of non-*E. coli Enterobacteriaceae*. These isolates were subcultured on MHA medium and identified using a combination of biochemical and molecular techniques. The consensus identification results for the clinical *Enterobacteriaceae* isolates are presented in Table 1 and the complete identification results are available in Additional file 2: Table S2. The consensus identification results revealed that the largest number of non-*E. coli Enterobacteriaceae* isolates were *Citrobacter freundii* (*n* = 22), followed by *Enterobacter cloacae* and *Klebsiella pneumoniae* (*n* = 15 isolates each) and *Escherichia hermannii* (*n* = 1). We were not able to identify unequivocally the genus of four non-*E. coli Enterobacteriaceae* isolates (SL184, SL191, SL201 and SL204) due to divergent phenotype identification results and ambiguous results of 16S rRNA and *rpoB* sequence based analysis. The sequence-based identification indicated that these four isolates belong to a non-*E. cloacae* species of *Enterobacter* or to *Leclercia* sp. (data not shown). This group of four isolates is therefore referred to as “*Enterobacter* sp./*Leclercia* sp.” All 13 isolates producing dark pink to reddish colonies included in the study were confirmed as *E. coli*.

### *Enterobacteriaceae* in the hospital environment

To explore the role of fomites as reservoirs or agents in the transmission of MDR *Enterobacteriaceae,* hospital surfaces were also examined via swab and culture. An initial set of 95 samples, collected between January 12th and 15th, 2014, resulted in 30 isolates producing dark blue colonies on CHROMagar Orientation medium. However, subsequent subculture, biochemical analyses, and 16S rRNA gene sequencing revealed that only four of these 30 isolates belonged to the *Enterobacteriaceae*. The remaining isolates were identified as *Bacillus* sp. (*n* = 23) and *Staphylococcus* sp. (*n* = 3), Additional file 2: Table S2. The analysis of an additional set of 94 hospital environmental samples, collected between March 31st and April 29th, 2014, yielded only one additional *Enterobacteriaceae* isolate, totaling only five *Enterobacteriaceae* isolates among the 189 environmental samples analyzed (2.7 %). Species-level identifications of these five isolates revealed two *K. pneumoniae*, two *E. cloacae* and one *Pantoea dispersa*.

### Urine isolate antibiotic susceptibility

The 70 urine isolates were tested for their susceptibility to 11 antibiotics or antibiotic combinations covering six classes of antimicrobial compounds and an ESBL phenotype (Table 1). Overall, 85.7 % of the clinical isolates were identified as MDR. More than 90 % of the urinary isolates were resistant to sulphonamides. Large proportions of the isolates were also found to be resistant to gentamycin (72.9 %), chloramphenicol (72.9 %), ampicillin/sulbactam (51.4 %), and/or ciprofloxacin (47.1 %). A considerable proportion of the clinical isolates (38.6 %) also demonstrated high resistance to the third generation cephalosporin, ceftazidime, which is typically indicative of ESBL production but may be due to inducible AmpC enzymes common in *C. freundii* and *Enterobacter* spp. Indeed, testing for the presence of ESBLs revealed that 64.3 % of the urine isolates produced an ESBL as defined by CLSI criteria. PCR-based detection of *bla*_CTX-M_ genes in every isolate presenting an ESBL phenotype (results not shown) confirmed the widespread presence of these genes and potentially enzymes in the analyzed isolate collection. In spite of the overall non-susceptibility to the majority of antimicrobials tested, none of the strains were resistant to tigecycline, and only two strains were resistant to imipenem. However, the two strains resistant to imipenem by disk diffusion – as well as all classified as having intermediate sensitivity - were deemed susceptible by CLSI standards when MICs were determined by E-test.

There appeared to be substantial species-specific differences in antimicrobial resistance with *C. freundii* being the most highly MDR species followed by *Enterobacter* sp./*Leclercia* sp., *E. hermannii*, *E. cloacae*, *K. pneumoniae* and *E. coli* (Table 2). All of the *C. freundii* urine isolates were resistant to sulfisoxazole, gentamycin and ciprofloxacin and most were resistant to several other antibiotics: the average *C. freundii* isolate was resistant to >7 antibiotics out of the 11 tested. In addition, 81.8 % of the *C. freundii* isolates produced ESBLs. The presence or absence of ESBLs in four isolates (SL129, SL154, SL155 and SL194) could not be determined based on phenotype only. Double disk synergy tests indicated the possible presence of an inhibitor-resistant ß-lactamase in these strains (data not shown).

**Table 2:** Percent prevalence of antimicrobial sensitivity phenotypes in urine isolates as determined by the disk diffusion method

Species	No.	Phenotype	Antibiotic^a^											ESBL
			ATM	CAZ	CIP	IMP	TGC	C	D	G	GM	K	SAM	
*Citrobacter freundii*	22	R	95.5	68.2	100.0	0.0	0.0	68.2	77.3	100.0	100.0	45.5	81.8	81.8
		I	0.0	31.8	0.0	22.7	40.9	4.5	22.7	0.0	0.0	50.0	18.2	
		S	4.5	0.0	0.0	77.3	59.1	27.3	0.0	0.0	0.0	4.5	0.0	
*Enterobacter* sp./*Leclercia* sp.	4	R	50.0	75.0	75.0	50.0	0.0	100.0	25.0	100.0	100.0	50.0	0.0	100.0
		I	50.0	25.0	25.0	25.0	75.0	0.0	0.0	0.0	0.0	50.0	100.0	
		S	0.0	0.0	0.0	25.0	25.0	0.0	75.0	0.0	0.0	0.0	0.0	
*Escherichia hermannii*	1	R	100.0	100.0	0.0	0.0	0.0	100.0	0.0	100.0	100.0	0.0	100.0	100.0
		I	0.0	0.0	100.0	0.0	0.0	0.0	0.0	0.0	0.0	100.0	0.0	
		S	0.0	0.0	0.0	100.0	100.0	0.0	100.0	0.0	0.0	0.0	0.0	
*Enterobacter cloacae*	15	R	46.7	33.3	6.7	0.0	0.0	93.3	6.7	100.0	93.3	26.7	46.7	86.7
		I	26.7	40.0	60.0	20.0	6.7	0.0	26.7	0.0	0.0	60.0	40.0	
		S	26.7	26.7	33.3	80.0	93.3	6.7	66.7	0.0	6.7	13.3	13.3	
*Klebsiella pneumoniae*	15	R	20.0	20.0	26.7	0.0	0.0	66.7	46.7	80.0	60.0	26.7	40.0	60.0
		I	20.0	20.0	46.7	26.7	26.7	0.0	20.0	0.0	0.0	20.0	26.7	
		S	60.0	60.0	26.7	73.3	73.3	33.3	33.3	20.0	40.0	53.3	33.3	
*Escherichia coli*	13	R	7.7	0.0	23.1	0.0	0.0	53.8	46.2	76.9	7.7	0.0	30.8	0.0
		I	0.0	0.0	7.7	15.4	0.0	0.0	23.1	0.0	7.7	0.0	15.4	
		S	92.3	100.0	69.2	84.6	100.0	46.2	30.8	23.1	84.6	100.0	53.8	
Overall	70	R	50.0	38.6	47.1	2.9	0.0	72.9	45.7	91.4	72.9	28.6	51.4	62.9
		I	12.9	24.3	27.1	21.4	24.3	1.4	21.4	0.0	1.4	37.1	28.6	
		S	37.1	37.1	25.7	75.7	75.7	25.7	32.9	8.6	25.7	34.3	20.0	

^a^Disk diffusion testing using the following antibiotics: *ATM* aztreonam, *CAZ* ceftazidime, *CIP* ciprofloxacin, *IMP* imipenem, *TGC* tigecycline, *C* chloramphenicol, *D* doxycycline, *G* sulfisoxazole, *GM* gentamicin, *K* kanamycin, *SAM* ampicillin/sulbactam. *R* resistant, *I* intermediate, and *S* sensitive phenotypes were determined based on CLSI criteria

Fourteen of the 15 *E. cloacae* isolates (93.3 %) were MDR, with 13 producing an ESBL and showing resistance for sulfisoxazole and a diminished sensitivity to at least five other antibiotics (with chloramphenicol, gentamycin, and kanamycin being among the most prevalent). The majority of *K. pneumoniae* isolates (73.3 %) were also MDR; most were insensitive to at least five antibiotics including chloramphenicol, doxycycline, gentamicin and a combination of ampicillin with sulbactam. Over half exhibited an ESBL phenotype, but a significant proportion (26.7 %) were sensitive to ciprofloxacin. All four *Enterobacter* sp./*Leclercia* sp. strains that were isolated from the urine cultures were positive for ESBL production and non-susceptible to eight or more antibiotics. One strain tested resistant or intermediate to all 11 antibiotics tested by disk diffusion. However, imipenem resistance and intermediate resistance in these isolates were not confirmed by E-test. The single *E. hermannii* identified among the urinary isolates presented a phenotype similar to the *Enterobacter* sp./*Leclercia* sp.: it exhibited ESBL production and resistance or intermediate resistance to all of the tested antibiotics with the exception of doxycycline, imipenem and tigecycline. Surprisingly, the *E. coli* strains were found to be the least resistant of all of the urine isolates tested and did not demonstrate the ESBL phenotype. Nevertheless, 61.5 % of the *E. coli* isolates could be classified as MDR and were most commonly resistant to sulfisoxazole, chloramphenicol and doxycycline.

### Hospital environment isolate antibiotic susceptibility

The same panel of 11 antibiotics were used for susceptibility testing of the five *Enterobacteriaceae* and 26 non-*Enterobacteriaceae* isolates from hospital fomites (Table 1). Two of the five *Enterobacteriaceae* isolates (*K. pneumoniae* SL513 and *E. cloacae* SL532) were MDR and carried ESBLs (Table 1). Of the three other *Enterobacteriaceae* isolates, *E. cloacae* SL512 was resistant to only sulfisoxazole and doxycycline and was sensitive to all of the other antibiotics tested. The remaining two isolates, *K. pneumoniae* SL531 and *Pantoea dispersa* SL552, were sensitive to all of the antibiotics tested.

Breakpoint thresholds have been published for *Staphylococcus* spp. for only seven of the 11 antibiotics used for susceptibility testing and for none of the 11 antibiotics used for testing *Bacillus* spp. For this reason, only the zone diameters of the 26 non-*Enterobacteriaceae* isolates identified as *Staphylococcus* spp. and *Bacillus* spp. are shown in Additional file 3: Table S3. Of the seven antibiotics for which *Staphylococcus* sp. breakpoints have been published, the tested *Staphylococcus* isolates were all deemed susceptible.

## Discussion

*E. coli* typically account for ~80 % of the bacteria isolated from uncomplicated UTI urines, with non-*E. coli Enterobacteriaceae* (e.g., *Klebsiella* spp*.*, *Enterococcus* spp.*, Citrobacter* spp.) usually present at much lower levels [27, 28]. This study was specifically designed to analyze the resistance patterns of non-*E. coli Enterobacteriaceae*. While we did not determine the overall prevalence of *E. coli* in the cultured urine samples, we collected all non-*E. coli Enterobacteriaceae* and were surprised to find that they were present in such a large proportion of the tested samples (57.0 %). Indeed, both *K. pneumoniae* and *E. cloacae* were cultured from 16.1 % of the urine samples - levels more typical of complicated UTIs or diabetic patients than general outpatient populations [28, 29]. It was particularly unexpected that *C. freundii* was isolated from almost a quarter (23.6 %) of the collected urine samples, accounting for 38.6 % of all non-*E.coli* isolates. While surprising, our findings are in agreement with a number of recent studies that have reported on the increased incidence of *Citrobacter* among urinary pathogens in developing countries [30–32], thus suggesting that *Citrobacter* may be becoming an increasingly important emerging urinary tract pathogen in resource-limited settings [33]. The high percentage of the non-*E. coli* UTI-associated bacteria analyzed in this study may also indicate weakened immune systems or additional health problems in the studied population. However, it is important to bear in mind that due to the relatively short duration of the study and small size of the sample, the observed proportions of pathogens recovered from urine samples may not be representative for the wider population of Bo and not be typical over longer periods of time.

Overall, data on antibiotic resistance in the *Enterobacteriaceae* from countries in West Africa (with the great majority originating from Nigeria and Senegal) are scarce and show enormous variation depending on the particular population and study design [12, 34]. For example, the prevalence of the ESBL phenotype in *Enterobacteriaceae* have been reported to vary from 10 % in diarrheal samples in Senegal to 96 % in Mali [34]. With respect to Sierra Leone however, there is an almost complete lack of information on bacterial antibiotic resistance.

The analysis of urine isolates used in this study suggests that clinically relevant MDR bacteria are prevalent in the studied outpatient population. Especially concerning is the high percentage of ESBL carriage among the analyzed non-*E. coli Enterobacteriaceae*, confirmed by both phenotypic and molecular assays. The chemotherapeutic options for the treatment of infections caused by these currently circulating *Enterobacteriaceae* strains are limited. Especially worrisome is the fact that a large proportion of these strains were not only resistant to first-line antibiotics such as sulphonamides (trimethoprim-sulfonamide is among the most commonly used treatments for uncomplicated UTIs [35]), ß-lactams and chloramphenicol, but many of them were also resistant to such second-line drugs as aminoglycosides and broad spectrum antibiotics such as the fluoroquinolone ciprofloxacin. While the high percentage of MDR isolates observed in this collection may be partly explained by the study design and overrepresentation of species rich in intrinsic resistance mechanisms (e.g. *Citrobacter* sp., *Enterobacter* sp.), even the usually less multiresistant species such as *E. coli* were found to contain significant proportion of MDR strains. On the other hand, despite earlier reports describing the detection of carbapenemase (i.e., *bla*_VIM-1_, *bla*_DIM-1_, *bla*_OXA-51_ and *bla*_OXA-58_) and tigecycline (*tet(X)*) resistance genes in this community [14, 36], there was no evidence for the spread of phenotypic resistance to these last-line antibiotics.

The contamination of the hospital surfaces is usually considered in the context of increased risk for hospital acquired UTIs and spread between inpatients or healthcare workers and patients. Mercy Hospital serves mostly the needs of an outpatient population (30–50 patients per day) with a limited number of inpatients (24 beds with varying occupancy). Many outpatients who spend considerable amounts of time in waiting rooms and other areas of the hospital, are exposed to hospital fomites and have contact with staff serving both outpatient and inpatient populations. This study made an attempt to determine the role of hospital environment fomites in the spread of MDR *Enterobacteriaceae* in Mercy Hospital. Previous studies have shown that bacterial pathogens, including members of *Enterobacteriaceae* such as *E. coli* and *K. pneumoniae,* are capable of surviving from days to over a year on dry, inanimate surfaces [37, 38]. Screening the environmental samples collected at Mercy Hospital demonstrated that the majority of isolates producing dark blue colonies on the chromogenic medium were *Bacillus* sp. Only five of the isolates collected from hospital surfaces (2.7 %) were identified as members of the *Enterobacteriaceae*. These results suggest that fomites in the Mercy Hospital open air ventilated environment are not a major source of pathogenic *Enterobacteriaceae* and that the observed resistance among the outpatient urinary isolates was likely not acquired from hospital fomite reservoirs. However, while relatively rare in the hospital environment, some of these environmental isolates demonstrated MDR phenotype and carried ESBLs. These observations warrant vigilance and the implementation of hospital hygiene procedures to maintain low levels of fomite contamination and potentially prevent the wider spread of these MDR pathogens.

## Conclusions

The surprisingly high incidence of *Citrobacter* found among urine isolates in the analyzed Mercy Hospital outpatient population suggests that *Citrobacter* may be becoming an increasingly important emerging urinary tract pathogen in this particular resource-limited setting. The high percentage of other non-*E. coli Enterobacteriaceae* among pathogens found in the urine of these patients may also indicate the prevalence of additional health problems in the studied population. Antibiotic susceptibility testing results show that clinically relevant MDR *Enterobacteriaceae* are prevalent in the studied population, which may reflect the lack of the national antibiotic stewardship policies in Sierra Leone and widespread access to antimicrobials without prescription. The results of hospital environment sampling showing very low level of contamination with MDR *Enterobacteriaceae* suggest that fomites are not a likely source of these pathogens in Mercy Hospital.

### Ethics approval and consent to participate

While there are no Human Subject Regulations in Sierra Leone, all the samples used in this study were collected in a bigger study approved by both Sierra Leone Ethics and Scientific Review Committee and the Njala University Institutional Review Board. All participants of that study or their parents/guardians provided informed written or verbal consent to be included in the study. As pre-existing diagnostic samples, these isolates were stripped of all personal identifiers and exempt from consideration as Human Subject Research as defined by 32 CFR 219, Section 101b in the US.

### Consent for publication

Not applicable.

### Availability of data and materials

All the data discussed in this manuscript are included in the manuscript or the accompanying additional/supplementary files. All the described bacterial isolates are available from the authors of the manuscript upon request and in compliance with local and international regulations related to transferring infectious biological samples.

## Additional files

Additional file 1: Table S1. Hospital environment sampling information. Information on the sources and dates of collection of hospital environmental samples. (XLS 30 kb) Additional file 2: Table S2. Full results of isolate identification. The full results of identification of isolates included in this study, including full results of phenotypic and molecular identification assays. (XLSX 51 kb) Additional file 3: Table S3. Sensitivity testing results of non-*Enterobacteriaceae* environmental isolates. Results of sensitivity testing of environmental isolates other than *Enterobacteriaceae* using disk diffusion method. (XLSX 62 kb)

## Abbreviations

ATM: aztreonam
C: chloramphenicol
CAZ: ceftazidime
CIP: ciprofloxacin
CLSI: Clinical and Laboratory Standards Institute
CRE: carbapenem resistant *Enterobacteriaceae*
CT/CTL: cefotaxime/cefotaxime + clavulanic acid
D: doxycycline
ESBL: extended spectrum β-lactamase
G: sulfisoxazole
GM: gentamicin
IMP: imipenem
K: kanamycin
MDR: multi-drug resistant
MHA: Mueller Hinton agar
MIC: minimum inhibitory concentration
PCR: polymerase chain reaction
SAM: ampicillin with sulbactam
TGC: tigecycline
TZ/TZL: ceftazidime/ceftazidime + clavulanic acid
UTI: urinary tract infection
